# Assessment of Effects of Pre- and Post-training Programme for Healthcare Professionals about Breastfeeding

**Published:** 2007-09

**Authors:** Ruhuşen Kutlu, Fatih Kara, Yasemin Durduran, Kamile Marakoglu, Selma Çivi

**Affiliations:** 1 Department of Family Physician, Meram Medical Faculty, University of Selçuk, Konya, Turkey; 2 Provincial Health Administration, Konya, Turkey

**Keywords:** Breastfeeding, Impact studies, Retrospective Studies, Training, Turkey

## Abstract

This retrospective study assessed the effects of pre- and post-training programme for healthcare professionals about breastfeeding. The study included 3,114 mothers who had children aged 1–72 month(s). Their knowledge and behaviours relating to breastfeeding were evaluated. The mothers were randomly divided into two groups: the ‘before’ group included 2,000 women who were not informed about breastfeeding, and the ‘after’ group comprised 1,114 women who had been informed about breastfeeding. 56.2% and 66.1% of the mothers started breastfeeding within 30 minutes after delivery, respectively, in the before and the after group (x^2^=29.31, p<0.001). 16.7% and 36.5% gave exclusive breastfeeding for six months (x^2^=72.85, p<0.001), and 28.5% and 23.7% stopped breastfeeding within the first five months (x^2^=17.20, p=0.002). Ninety-four percent delivered in a hospital or in a primary healthcare centre. Therefore, prenatal and postnatal breastfeeding education and support courses may improve a woman's chance of starting and continuing to breastfeed her baby. In terms of the number of antenatal check-ups, since the differences between the two groups were significant (x^2^=390.67, p=0.000), the importance of the training programme about breastfeeding was highlighted. Follow-up interventions after training are suggested.

## INTRODUCTION

Benefits of breastfeeding for both mothers and babies have been well-documented (1). Breastfed babies are at a significantly reduced risk of gastrointestinal, urinary and respiratory infections, and breastfeeding enhances neurodevelopment, especially in premature infants. Breastfeeding also protects women from developing postpartum haemorrhage, breast cancer, and ovarian cancer (2).

Recognizing the health implications of appropriate breastfeeding practices, various breastfeeding-promotion and support strategies have been established worldwide with varying effects (3). This concern was reflected on a global scale when the World Health Organization (WHO) and United Nations Children's Fund (UNICEF) launched the Baby-Friendly Initiative (BFI) in 1991, in response to the Innocenti Declaration 1990. Both physicians and nurses can significantly influence the knowledge, attitudes, and practices of mothers on the initiation and maintenance of breastfeeding, if health professionals themselves have adequate knowledge on the benefits of breastfeeding, a positive attitude, and the necessary clinical management skills for counselling (4).

In mid-2002, the Provincial Health Administration in Konya city organized a three-day training on the WHO/UNICEF guidelines of breastfeeding for all chiefs of paediatrics and gynaecological units in the state, the army, private and university hospitals. It was perceived that, after the training, these doctors would train the healthcare staff in their hospitals. In this context, we, the training team of this research, evaluated the breastfeeding knowledge and behaviours of mothers before and after the implementation of the Baby-Friendly Hospital Initiative (BFHI) in Konya, Turkey. The main objective of the study was to evaluate whether the healthcare professionals changed their care practices with regard to provide breastfeeding information or support.

## MATERIALS AND METHODS

This is a retrospective review of breastfeeding outcomes at delivery-sites before and after a specific intervention of lactation education of health professionals. The study, organized by the Provincial Health Administration, was conducted during 15 June 2002–20 February 2004. Before 2002, the BFHI was unknown. In 2003, paediatricians, gynaecologists, family doctors, and nurses participated in a course on appropriate breastfeeding and the principles of BFHI. We evaluated the knowledge and behaviours of 3,114 mothers relating to the first initiation, duration of, weaning time, and factors affecting breastfeeding. Exclusively-breastfed infants received only breastmilk and no other liquids or solids, except vitamins, mineral supplements, or medicines. Weaning time means giving up ‘breastfeeding’ completely.

The participants were randomly chosen from among mothers who had babies aged 1–72 month(s) and who came to a medical institution for any reason. They were randomly divided into two groups. The first group, comprising 2,000 women, never attended a training programme on breastfeeding (the before group), and the second group comprised 1,114 women who participated in the training programme (the after group). Women were asked about their opinions about breastfeeding before the training of health staff. Then, all the health personnel participated in an educational programme related to supporting and protecting breastfeeding. The trained health personnel informed the mothers in the second group about breastfeeding after their delivery or during the vaccination of their babies.

The chi-square tests were used for assessing the statistical significance between the two groups. The p value of <0.05 was considered significant.

## RESULTS

The mean ages of the mothers in the before group and the after group were 26.90 (SD±5.57) and 26.81 (SD±7.13) years respectively. Their age composition was similar (p>0.05). Table 1 presents their sociodemographic characteristics. Both the groups were comparable in terms of prenatal characteristics and the place of delivery (Table 2). Table 3 summarizes the breastfeeding characteristics. 56.2% and 66.1% of the mothers started breastfeeding in the first 30 minutes after delivery, respectively, in the before and the after group (x^2^=29.31, p<0.001). 16.7% and 36.5% breastfed exclusively for six months (x^2^=72.85, p<0.001). Breastfeeding was terminated within the first five months respectively by 28.5% and 23.7% of them (x^2^=17.20, p=0.002). The percentage of mothers using an artificial nipple was higher in the before group (51.8%) than that in the after group (41.1%) (x^2^=42.76, p<0.001).

**Table 1.** Sociodemographic characteristics of mothers

Sociodemographic characteristics	Before group		After group		*x*^2^	p value
	No.	%	No.	%		
Education∗						
Illiteracy	152	7.6	97	8.7		
Primary school	1,308	65.5	719	64.5		
Middle and high school	380	19.0	203	18.2		
University	156	7.8	94	8.4	1.31	0.252
Employment†						
Housewife	1,795	89.7	995	89.3		
White-collar	180	9.0	116	10.4		
Blue-collar worker	25	1.3	3	0.3	5.10	0.024
Living with husband‡						
Yes	434	21.8	208	18.7		
No	1,565	78.2	899	80.3	21.01	<0.001
Sharing the same house with a grandparent∗∗						
Yes	783	39.2	443	40.0		
No	1,215	60.8	665	60.0	0.18	0.665

∗ 5 persons did not answer this question;

† 3 persons did not answer this question;

‡ 7 persons did not answer this question;

∗∗ 8 persons did not answer this question

**Table 2.** Prenatal characteristics of mothers

Prenatal characteristics	Before group		After group		*x*^2^	p value
	No.	%	No.	%		
Number of antenatal check-ups∗						
None	178	10.1	45	4.0		
2–3	609	34.4	65	5.8		
4–6	523	29.5	511	45.9		
7 and more	460	26.0	493	44.3	390.67	0.000
Place of delivery†						
Home, self-delivery	134	6.7	67	6.0		
Primary healthcare centre	209	10.5	97	8.7		
Hospital	1,655	82.8	944	85.3	3.06	0.216
Type of delivery‡						
Natural	1,685	84.5	902	81.7		
Cesarean	308	15.5	202	18.3	4.17	0.041

∗ 230 persons did not answer this question;

† 8 persons did not answer this question;

‡ 17 persons did not answer this question

**Table 3.** Characteristics of breastfeeding

Characteristics	Before group		After group		*x*^2^	p value
	No.	%	No.	%		
Initiation of breastfeeding						
Within 30 minutes after delivery	1,124	56.2	736	66.1		
Within 1–2 hour(s) after delivery	594	29.7	263	23.6		
Later	282	14.1	115	10.3	29.31	<0.001
Type of breastfeeding						
On demand	1,794	89.7	1,033	92.7		
Scheduled	206	10.3	81	7.3	7.84	0.005
Duration (months) of exclusive breastfeeding						
0–5	631	77.8	298	56.7		
6	135	16.7	192	36.5		
7 and over	45	5.5	36	6.8	72.85	<0.001
Artificial nipple						
Yes	1,036	51.8	458	41.1		
No	904	46.6	656	58.9	42.76	<0.001
Weaning (months) of breastfeeding†‡						
0–6	570	28.5	253	23.7		
7–11	508	25.4	316	29.6		
12–17	400	20.0	247	23.1		
18–23	360	18.0	162	15.2		
24 and over	162	8.1	91	8.4	17.20	0.002

∗ 60 persons did not answer this question;

† Current breastfeeding was not included;

‡ 45 persons did not answer this question

## DISCUSSION

Generally, breastfeeding is widespread, and family members in Turkey support it. Recently, under the management of the Provincial Health Administration, the healthcare personnel in the hospitals were trained. Prenatal support, hospital management, and subsequent paediatric and maternal visits are all important components of breastfeeding promotion (4).

In our study, most (92.2%) mothers were at middle/low educational level and were housewives. The rates of seven and more antenatal check-ups in the two groups were 26.0% and 44.3% respectively. This ratio was statistically higher in the second group than in the first group (x^2^=390.67, p<0.001). Because of the mother's instinct, the important experiences relating to rearing a baby, especially breastfeeding, are kept in mind and re-applied for their future babies. The rates of time of initiating breastfeeding within 30 minutes of delivery were 56.2% and 66.1% in the before and the after group respectively. The rate of breastfeeding initiation within the first 1–2 hour(s) of normal spontaneous delivery significantly showed a higher prevalence than cesarean section (p<0.001). Cesarean delivery had a negative effect on early and successful breastfeeding (4). Dewan et al. recommend that an attempt must be made to initiate lactation before the end of the first hour of birth following cesarean delivery (4). According to Merewood, in 2001, the mean initiation rate of breastfeeding in US baby-friendly hospitals was 84.8% compared to a national breastfeeding initiation rate of 69.5% (5). This rate is higher than that in our study.

When we asked about the type of breastfeeding, we found that the rates of breastfeeding whenever the baby demanded in the two groups were 89.7% and 92.7% respectively. The rates of breastfeeding with scheduled periods (i.e. every three hours or at fixed times whenever the mother plans) were 10.3% and 7.3% respectively. These findings are comparable with the recommended breastfeeding practices of WHO (6,7). In this study, the rates of exclusive breastfeeding for six months in the two groups were 16.7% and 36.5% respectively. The second rate was significantly higher than the first rate (x^2^=72.85, p<0.001). Lack of knowledge and failure of training programmes decrease the rates of exclusive breastfeeding (8). According to Kramer et al., the exclusive breastfeeding rate at sixth months in the Republic of Belarus in 1997 was 7.9% (8). Durand M et al. reported that exclusive breastfeeding rates of a pre– and post–sample survey were 14.0% and 28.0% respectively. In the same study, the initiation rate of breastfeeding was 76.0% (9). In our study, this ratio was also significantly different (16.7% vs 36.5%) in the after group compared to the before group.

Before the BFHI project, there was no room for breastfeeding at the hospitals in Konya. Mothers breastfed and changed the diapers of their babies in the hospital's hall and in front of the polyclinics. Implementation of the project resulted in many rooms for breastfeeding purposes in all the health facilities.

The rates of using artificial nipples in the two groups were 53.4% and 41.1% respectively. This ratio was significantly lower in the second group than in the first group. The use of an artificial nipple is an unhealthy habit (10). Unfortunately, the before group had a great tendency to use an artificial nipple.

Moreover, sharing of the same house with grandparents is a Turkish traditional custom; approximately 40% share the same house. Having a grandparent in the same house positively supports and encourages breastfeeding as an ideal form of nutrition for infants.

In our study, the weaning rate of breastfeeding within six months was significantly lower in the after group than that in the before group (x^2^=17.20, p=0.002). As a result, breastfeeding knowledge, training experiences, and activities of healthcare professionals encouraged and helped women successfully to initiate and continue breastfeeding their babies.

Our results demonstrate that successful implementation of baby-friendly policies was associated with an increase in the rates of breastfeeding education. Breastfeeding knowledge, attitudes, and practices among healthcare professionals should be improved worldwide. Finally, if all mothers and all health personnel are aware of the benefits of breastfeeding, the percentage of improved health of children will increase.
